# Factors associated with inadequate prenatal care service utilization in Ethiopia according to the WHO recommended standard guidelines

**DOI:** 10.3389/fpubh.2022.998055

**Published:** 2022-11-03

**Authors:** Berhanu Teshome Woldeamanuel

**Affiliations:** Department of Epidemiology and Biostatistics, School of Public Health, Saint Paul's Hospital Millennium Medical College, Addis Ababa, Ethiopia

**Keywords:** prenatal care, adequacy, Ethiopia, multilevel analysis, WHO recommended standard guidelines

## Abstract

**Background:**

Adequate maternal health care could prevent 54% of maternal deaths in low- and middle-income countries. In Ethiopia, the maternal mortality rate was reduced from 817 to 412 deaths per 100,000 live births between 2000 and 2016. Thus, the current study focuses on the adequacy of prenatal care (PNC) services rather than the mere prenatal contacts available to assess compliance with the WHO recommended standard guidelines.

**Methods:**

A nationally representative cross-sectional dataset from the Ethiopian Mini Demographic and Health Survey 2019 was analyzed. Risk factors for prenatal care adequacy were assessed using a multilevel ordinal logistic regression model.

**Results:**

About 43% of women met the old WHO recommendation of at least four prenatal contacts, while only 3.5% of women met the new WHO recommended minimum of eight prenatal contacts. The overall adequacy of prenatal care based on the four prenatal care utilization indicators was 52.1% no PNC, 37.4% received inadequate PNC and 10.5% received adequate PNC. Being a rural resident [AOR = 0.694 (95% CI: 0.557, 0.865)] and wanting no more children [AOR = 0.687 (95% CI: 0.544, 0.868)] are associated with inadequate prenatal care. Higher educational attainment of women and spouses, exposure to the media, upper wealth quintile, and a perceived shorter distance to a health facility were significantly associated with adequate prenatal care.

**Conclusion:**

The prevalence of adequate prenatal care was lower. Multi-sectoral efforts are needed to improve maternal health targets by reducing maternal mortality through improved health care services.

## Introduction

Maternal mortality is a major issue in sub-Saharan Africa (1). Following the end of the Millennium Development Goals (MDGs) in 2016, the World Health Organization (WHO) recommends focused prenatal care from a skilled provider comprising a minimum of eight contacts, with the first contact made within the first trimester, and the necessary contents of prenatal care, to improve women's experience of care and reduce maternal and perinatal mortality (2). Although the global maternal mortality rate declined by 38% from 342 to 211 deaths per 100,000 live births between 2000 and 2017, about 94% of maternal mortality occurred in low and middle-income countries. The sub-Saharan Africa region accounted for 62% of global mortalities, with a maternal mortality rate of 510 deaths per 100,000 live births (3). In Africa and other developing nations, pregnancy complications are the main cause of death among mothers aged 15–49 years. These countries account for over two-thirds of the approximately 300,000 preventable deaths globally each year during pregnancy and delivery. Improved quality of health care during pregnancy and childbirth can prevent nearly 75% of maternal deaths in poor countries; adequate antenatal care can prevent 26%, and increased access to quality obstetric care can prevent 48% (4). This demonstrates that improved prenatal coverage and high-quality prenatal service delivery are unquestionably required to meet SGDs after 10 years.

Prenatal care (PNC) is a vital component of continuum care for mothers and babies, achieved through early diagnosis of obstetric conditions, detection of complications arising during pregnancy, women's education about the danger signs of pregnancy, and the benefits of breastfeeding (5). Increased coverage and quality of maternal health care could prevent 71% of neonatal deaths, 33% of stillbirths, and 54% of maternal deaths in low-and-middle-income countries (6). Despite a very high maternal mortality rate, Ethiopia has been making steady progress in reducing the maternal mortality rate (MMR) from 817 deaths per 100,000 live births in 2000 to 412 deaths per 100,000 live births in 2016 (7). Recognizing the importance of prenatal care, the Ethiopian government endorsed the 2015 Health Sector Transformation Plan (HSTP) (8), which targets to reduce the MMR to 199 deaths per 100,000 live births and the neonatal mortality rate to 10 per 1,000 live births by 2020, emphasizing providing quality prenatal care. Despite these policies and intervention efforts, the progress made so far has not been enough to achieve the above goals. For instance, the 2019 interim Demographic and Health Survey (DHS) estimate of the neonatal mortality rate was more than threefold (33 per 1,000 live births) higher compared to the HSTP target (9).

The use of health facilities is significantly related to prenatal care contacts, and adequate prenatal care entails both the use of services and the sufficiency of the content within the services (10, 11). Prenatal care is critical for improving maternal and neonatal health (2). In 2019, 74% of women who gave birth in Ethiopia received prenatal care from a skilled provider at least once, and despite four or more ANC visits (43%) from skilled providers, only 20% of pregnant women had their first ANC during the first trimester in 2016. Furthermore, concerning the type of provider, doctors (5.7%), nurses/midwives (42%), health officers (1.4%), and health extension workers (13.2%), respectively, provided prenatal care (9).

Even though the percentage of women attending prenatal care has increased in Ethiopia, maternal and neonatal mortality remains high (7). Thus, to achieve Sustainable Development Goal 3, of universal health coverage and reducing MMR to <70 per 100,000 live births by 2030, the quality of the services needs to be addressed. The current study focused on the adequacy of prenatal care services rather than the mere prenatal contacts available to Ethiopian women to ensure the well-being of mothers, fetuses, and newborns using a recent nationally representative survey. The present study uses Donabedian's model of quality of care and includes prenatal care use by skilled or unskilled providers, timeliness, sufficiency, and appropriateness of prenatal care services (12). Further, Heredia et al. (13) introduced dimensions such as skilled care, timeliness, and appropriateness of care while measuring the prenatal care service adequacy available in Mexico. Beeckman et al. (14) used the content and timing of PNC in pregnancy to assess women's receipt of quality prenatal care. The present study uses these indicators to provide comprehensive data on the appropriateness and adequacy of prenatal care available to women in Ethiopia, taking into account various socioeconomic and demographic characteristics.

Previous research in Ethiopia and elsewhere found that rural residence, lower maternal and spouse education, lower household wealth index, higher birth order, and long distance from medical help were consistently associated with prenatal health care use (15–28). Addressing the quality of prenatal care is key to achieving the Sustainable Development Goals' targets of reducing maternal and newborn mortality. The utilization of prenatal care services was not optimal in terms of the frequency of contact as well as the coverage of items received. Adequate prenatal care includes access to prenatal care, the number of prenatal care contacts, and the content of prenatal care (11, 13). There may be problems with insufficient timeliness, such as a late start of prenatal care contacts, a lower than recommended number of contacts, or incomplete components received. The previous studies in Ethiopia never fully analyzed the quality of prenatal care service utilization, assessing compliance with the WHO recommended standard guidelines considering the skilled health care provider, timeliness, sufficiency of coverage, and appropriateness of prenatal content received. To the best of our knowledge, most previous research on maternal health care in Ethiopia focused on the timing and coverage of prenatal contacts. Increasing recognition of the role of poor-quality care in poor maternal and neonatal outcomes has stimulated interest in assessing the quality of maternal health services. There is little information on prenatal care service quality in Ethiopia and even less data on related determinants of prenatal care quality. Further, no study, based on the service provision assessment data at the national level, has examined multiple dimensions of quality of care.

## Methods

### Study design and data source

This study used secondary data from the 2019 Ethiopia Mini Demographic and Health Survey (EMDHS). The 2019 EMDHS is a nationally representative household survey that collects a very wide range of population, health, and other important indicators covering all nine regions and two administrative cities of Ethiopia. Participants in the survey were asked retrospective questions spanning 5 years before the survey.

### Sampling approach and study population

The 2019 Ethiopian mini DHS used a two-stage sample design to estimate important indicators at the national level, as well as in urban and rural areas of each administrative region. The first step was to choose a sample of clusters made up of enumeration areas (EAs) generated for the 2019 Ethiopian Population and Housing Census (EPHC), by the Central Statistical Agency (CSA). For the 2019 EPHC, a total of 149,093 EAs encompassing an average of 131 households were created. A total of 21 strata for sampling were created by dividing each region into urban and rural areas. Implicit stratification and proportional allocation were achieved at each of the lower administrative levels by sorting the sampling frame within each sampling stratum before sample selection, according to administrative units at various levels, and selecting a probability proportional to size at the first stage of sampling (9).

Twenty-five EAs were chosen from eight regions, and 35 EAs were chosen from each of the three major regions [Amhara, Oromia, and the Southern Nations, Nationalities, and Peoples' Region (SNNPR)] to ensure that survey precision was comparable across regions. A total of 305 EAs were chosen in the first stage, with a probability proportional to EA size (93 in urban areas and 212 in rural areas). From January to April 2019, a household listing operation was conducted in all designated EAs. Since some of the EAs chosen for the 2019 EMDHS were big, with more than 300 households, each large EA was segmented, and only one segment was chosen for the survey, with a probability proportionate to segment size. As a result, only the households in the specified segment were listed; that is, a 2019 EMDHS cluster is either an EA or a section of an EA. A specified number of 30 households per cluster were selected in the second step of the selection process, with an equal likelihood of systematic selection from the newly formed household listing. The current study looked at 7,174 women between the ages of 15 and 49 (9).

### Variables

#### Outcome variable

The outcome variable was prenatal care adequacy and measured using four prenatal care utilization indicators: skilled health care provider (doctors, nurses, midwives, health officers), timeliness (first PNC contact within the first trimester), the sufficiency of prenatal care contacts (at least four contacts during pregnancy), and appropriateness of core prenatal care contents received. Standard guidelines for prenatal care in Ethiopia recognize the following indicators of the appropriateness of prenatal care from a skilled provider: receiving iron supplements for at least 180 days; treatment for intestinal parasites; at least two doses of Tetanus Toxoid injections; blood pressure measurement; urine test; blood sample is taken to test for possible morbidities; nutritional counseling; health education on danger signs and complications during pregnancy; health education on birth preparation; and HIV/AIDS counseling, testing, and collection of results (29).

Referring to the previous studies, women who received at least seven of the ten contents above were considered to receive appropriate content in this study since globally, there is no common standard to categorize prenatal care contents as appropriate (13, 30). Thus, the outcome variable (prenatal care adequacy) is the composite index that comprises the above four indicators defined as 2 = received adequate prenatal care (care delivered by skilled health personnel, first prenatal contact within the first trimester, a sufficient number of prenatal contacts (4 or more) and received appropriate content, 1 = received inadequate prenatal care (care delivered to women who are not strictly adherent to the above criteria) and 0 = received no prenatal contact (where care was either not received by the woman or failed to adhere to even one of the criteria of adequate prenatal care).

#### Independent variables

The explanatory variables, adopted from various literature (15–28) include maternal age, woman and spouse education, employment status of mother and spouse, access to media (radio, television), household wealth index, women's autonomy using who decides respondents' health care access as a proxy (respondent alone, respondent/spouse, and spouse alone). Additionally, birth interval, birth order, children ever born, current marital status, and religion are used. Others are wanted child when pregnant, household headship, health insurance coverage, place of residence, and region. The DHS uses five wealth quintiles. Households are given scores based on the number and kinds of consumer goods they own, ranging from a television to a bicycle or car, in addition to housing characteristics such as the source of drinking water, toilet facilities, and flooring materials. These scores are derived using principal component analysis. National wealth quintiles are compiled by assigning the household score to each usual (de jure) household member, ranking each person in the household population by her or his score, and then dividing the distribution into five equal categories, each comprising 20% of the population. We created three community-level factors to find community characteristics that could affect PNC adequacy during pregnancy. The three factors are the community poverty rate (high or low), community illiteracy rate (high or low), and community media access rate (high or low) as the proportion of respondents within each community who are poor, illiterate, and with no media access, respectively.

The community-level variables included in the analysis were based on women's characteristics, particularly those that have implications for accessing prenatal care. Because the EMDHS did not collect aggregate level data at the community level, we used EAs to represent communities prominently. The community level factors are women's residential status (urban, rural), and geographical region (Tigrai, Afar, Amhara, Oromia, Somali, Benishangul-Gumuz, SNNPR, Gambela, Harari, Dire Dawa, Addis Ababa). Individual level attributes were aggregated at the cluster (primary sample unit) level to create community-level variables, which were then categorized as low or high based on the distribution of the proportion values generated for each community. The cutoff point for the categorization was the mean value if the aggregate variable was normally distributed, and if not, the median value was used. Community-level poverty was categorized as high if the proportion of women from the two lowest wealth quintiles in a given community was 43–100% and low if the proportion was 0–42%. Community-level media use was categorized as high if the proportion was 60–100% and low if the proportion of women who use media in the community was 0–59%. Community illiteracy was classified as high if the proportion of women who could not read at all was 67–100% and low if the proportion of women who could not read at all was 0–66%. Community-level women's autonomy was categorized as high if the proportion of women who had at least moderate autonomy was 61–100% and low if the proportion was between 0 and 60% (17–26).

### Statistical analysis

The statistical software Stata 14 was used to analyze the data (31). The data were weighted using sampling weight, primary sampling unit, and strata before any statistical analysis to restore the representativeness of the survey and take into account the sampling design when calculating standard errors. A Generalized Structural Equation Modeling (with the logit link function and a robust variance estimator for the standard error) approach was employed. The degree of unadjusted or crude association between the covariates and the outcome variable was assessed using the chi-square test. Since the EMDHS, 2019 follows a hierarchical data structure as the surveys are based on two-stage stratified cluster sampling (9), the multilevel ordinal logistic regression model was fitted to assess regional variation in adequacy of prenatal care and identify associated factors.

In the EMDHS 2019, women were nested within households, and households were further nested within clusters. Due to the hierarchical nature of this survey, a multilevel regression modeling approach ensures that household and cluster variations are properly accounted for to avoid parameter overestimation. In accounting for these variations, enumeration areas referred to as clusters were considered a level-2 variable, while those of respondents or individual-level variables were assigned level-1. Although we used a multilevel mixed-effects model because of the need to adjust and obtain parameter estimates through a fixed effects (multivariable) model, the nesting nature of the EMDHS data (multilevel) and the need to account for the cluster effects (*via* a random-effects approach), which are not included in the data set. When the variance of the residual errors is correlated between individual observations as a result of these nested structures, multilevel ordinal logistic regression was used to assess the relationship between levels of the adequacy of prenatal care and associated factors.

In multilevel regression, the clustering effect plays a great role in the estimation of the parameters, and this clustering effect can be quantified by intraclass correlation (ICC). ICC is the proportion of total variation in the response variable that is accounted for by between-group variation (32). In the current study, all predictors are at level 1, and the authors are interested in studying the effect of the clustering variable, which is the region where the women were dwelling. On the other hand, the categories of the response variable (prenatal care adequacy) are three, which are ordered as “no prenatal care”, “inadequate” and “adequate” and hence ordinal logistic regression has potentially greater power than that of binary logistic regression and the baseline-category logit models as it takes into account information on the order of values (33).

All the outputs for descriptive as well as fitting multilevel ordinal logistic regression analysis were done using weights provided in the EMDHS 2019 data as per the recommendations by the DHS program. The way weights are used varies based on the purpose of the analysis. The weights from DHS were used to carry out multilevel analysis and adjusted as per the recommendation by Adam (34). Subsequently, we checked the goodness of fit after weighting the dataset by both candidate weights.

### Model building with potential risk factors

Let *p*_*ij*_ denote the probability that the response variable is below or equals a particular category. *p*_*kij*_ = *P*(*y*_*ij*_ = *j*|*x*_*ij*_) the probability that *i*th woman received prenatal care in the *j*th region. The two-level logistic regression model can be given as:


$$logit\left(p_{kij}\right)=\log\left[\frac{P(y_{i}\leq k)}{P(y_{ij}>k)}\right]=\beta_{0}+\;\beta_{1}X_{ij}+U_{oj}\;$$

where *U*_*oj*_ is a random quantity and follows a normal distribution with mean zero and variance $\sigma_{u}^{2}.$ This model can be split into two models; one for level 1 and the other for level 2

$\begin{array}{l} \text{Level }1,\text{ random intercept model }:\;logit\left(p_{ij}\right)=\log\left[\frac{p\left(y_{ij\leq k}\right)}{p\left(y_{ij\geq k}\right)}\right]=\beta_{oj}+\beta_{1}X_{ij} \end{array}$

$\begin{array}{l} \text{Level }2,\text{ empty model}:\beta_{oj}=\beta_{o}+U_{oj} \end{array}$

Thus, $logit\left(P_{kij}\right)= log\left(\frac{p\left(y_{ij\leq k}\right)}{p\left(y_{ij\geq k}\right)}\right)=\beta_{o}+\overset{k}{\underset{h=1}{\sum }}\beta_{h}x_{ij}+U_{oj}+\overset{k}{\underset{h=1}{\sum }}U_{hj}x_{ij}\;$the random intercept multilevel ordinal logistic regression model, the first part${\;\beta }_{o}+\overset{k}{\underset{h=1}{\sum }}\beta_{h}x_{ij\;}$is called the fixed part of the model, and the second part, $U_{oj}+\overset{k}{\underset{h=1}{\sum }}U_{hj}x_{ij}$ is called the random part of the model.

The random variables or effects, *U*_0*j*_, *U*_1*j*_, …, *U*_*kj*_ are assumed to be independent between groups (regions) but may be correlated within groups (regions). Then the intra-class correlation (ICC) which quantifies the degree of heterogeneity of prenatal care between clusters (the proportion of the total observed variation in prenatal care that is attributable to cluster variations) at the region's level is given by (35):


(1) $$\begin{array}{l} ICC=\frac{{\sigma_{\mu }}^{2}}{{\sigma_{\mu }}^{2}+\pi^{2}/3}\; \end{array}$$

Where ${\sigma_{\mu }}^{2}$ is the between group variance.

Further, the Median Odds Ratio (MOR) quantifies the variation or heterogeneity in prenatal care between clusters in terms of the odds ratio is defined as (36):


(2) $$\begin{array}{l} MOR=\exp\left\{0.95\sigma_{\mu }\right\}\; \end{array}$$

The MOR measures the median value of the odds ratio between the cluster with a high likelihood of receiving adequate prenatal care and the cluster at a lower risk when randomly picking out individuals from two clusters (enumeration areas). It is a measure of unexplained cluster heterogeneity.

The specifications of the models were based on variables that showed significant associations in the bivariate multilevel proportional odds model analysis (*p*-values≤ 0.2). Four models were built for the multilevel analysis. Model 1 constituted a null model without predictor variables to assess the extent of cluster variation in prenatal care adequacy, and Model 2 was formulated with individual-level factors. Model 3 involves the community-level factors while Model 4 is fitted with both individual and community level factors. The multilevel modeling approach was used for all of these models. Model comparison was made based on deviance [−2Log-Likelihood Ratio (LLR)] since the models were nested, and the model with the lowest deviance was the best-fitted model for the data. Further, we have tested the proportional odds assumptions of constant effects of all independent variables across categories of the outcome variable using the Brant test.

### Ethical consideration

This study used publicly available Ethiopian Mini Demographic and Health Survey (EMDHS) data. Permission was obtained from the Measure DHS program to access and analyze the data. During EMDHS data collection, Informed consent was taken from each participant, all identifiers were removed and the confidentiality of the information was maintained.

## Results

### Characteristics of the participants

Only about 18% of women who were pregnant 5 years before the survey attended school beyond primary school, whereas about 40% didn't attend school. The majority of the respondents, about 67.8%, are rural residents. About 78% of the respondents are from male-headed households. Nearly half (56%) of the respondents are housewives and about 20% are engaged in agricultural work. About 65% of respondents have no access to mass media. Almost one-fourth (24.9%) of respondents were younger women (15–19), whereas about 15% of them were over 40 years old and the distribution is nearly uniform in other age groups.

Regarding women's autonomy in decision making, respondents' health care access as a proxy, more than half (58%) of both respondents and husbands, and only 16% of respondents alone. Most of the respondents (79.8%) wanted pregnancy then and only 6.4% wanted no more. Similarly, the majority (79.6%) of women have at least two children. Almost two-thirds (65%) of the respondents were married (Table 1).

**Table 1:** Descriptive characteristics of women in Ethiopia by adequacy of PNC received, 2019 interim Demographic and Health Survey.

			**PNC adequacy**		
			**No PNC**	**Inadequate PNC**	**Adequate PNC**
**Variables**	**Categories**	***n*** **(%)**	***n*** **(%)**	***n*** **(%)**	***n*** **(%)**
Birth order	First	1,466 (20.4)	565 (38.5)	636 (43.4)	265 (18.1)
	2–3	2,213 (30.8)	1,004 (45.4)	911 (41.1)	298 (13.5)
	4–5	1,632 (22.7)	935 (57.3)	588 (36.0)	109 (6.7)
	6+	1,863 (26.0)	1,233 (66.2)	548 (29.4)	82 (4.4)
Residence	Urban	1,507 (21.0)	280 (18.6)	812 (53.9)	415 (27.5)
	Rural	5,667 (79.0)	3,457 (61.0)	1,871 (33.0)	339 (6.0)
Wealth quantile	Poorest	2,424 (33.8)	1,808 (74.6)	535 (22.1)	81 (3.3)
	Poorer	1,178 (16.4)	665 (56.5)	450 (38.2)	63 (5.3)
	Middle	1,023 (14.3)	538 (52.6)	407 (39.8)	78 (7.6)
	Richer	915 (12.8)	423 (46.2)	402 (43.9)	90 (9.8)
	Richest	1,634 (22.8)	303 (18.5)	889 (54.4)	442 (27.1)
Mother education	No education	4,346 (60.6)	2,835 (65.2)	1,287 (29.6)	224 (5.2)
	Primary	1,938 (27.0)	760 (39.2)	921 (47.5)	257 (13.3)
	Secondary	575 (8.0)	113 (19.7)	310 (53.9)	152 (26.4)
	Higher	315 (4.4)	29 (9.2)	165 (52.4)	121 (38.4)
Woman Age	15–19	358 (5.0)	186 (52.0)	139 (38.8)	33 (9.2)
	20–24	1,490 (20.8)	741 (49.7)	585 (39.3)	164 (11.0)
	25–29	2,008 (28.0)	968 (48.2)	777 (38.7)	263 (13.1)
	30–34	1,527 (21.3)	793 (51.9)	585 (38.3)	149 (9.8)
	35–39	1,142 (15.9)	640 (56.0)	402 (35.2)	100 (8.8)
	40–44	482 (6.7)	298 (61.8)	149 (30.9)	35 (7.3)
	45–49	167 (2.3)	111 (66.5)	46 (27.5)	10 (6.0)
	at least once a week	1,053 (14.7)	148 (14.1)	572 (54.3)	333 (31.6)
Current pregnancy desired	Wanted then	5,725 (79.8)	2,985 (52.1)	2,116 (37.0)	624 (10.9)
	Wanted later	989 (13.8)	471 (47.6)	409 (41.4)	109 (11.0)
	Wanted no more	460 (6.4)	281 (61.1)	158 (34.3)	21 (4.6)
Spouse education	no education	3,128 (43.6)	2,105 (67.3)	867 (27.7)	156 (5.0)
	Primary	2,155 (30.0)	1,024 (47.5)	929 (43.1)	202 (9.4)
	Secondary	744 (10.4)	205 (27.6)	384 (51.6)	155 (20.8)
	Higher	566 (7.9)	131 (23.1)	268 (47.3)	167 (29.5)
Who decides respondent's healthcare	respondent alone	1,177 (16.4)	580 (49.3)	451 (38.3)	146 (12.4)
	respondent and husband	4,165 (58.1)	2,076 (49.8)	1,622 (38.9)	467 (11.2)
	husband alone	1,287 (17.9)	824 (64.0)	390 (30.3)	73 (5.7)
Distance	Big problem	3,790 (52.8)	2,397 (63.2)	1,161 (30.6)	232 (6.1)
	Not a big problem	3,384 (47.2)	1,340 (39.6)	1,522 (45.0)	522 (15.4)
Region	Tigrai	766 (10.7)	228 (29.8)	401 (52.3)	137 (17.9)
	Afar	645 (9.0)	477 (74.0)	148 (22.9)	20 (3.1)
	Amhara	763 (10.6)	413 (54.1)	268 (35.2)	82 (10.7)
	Oromia	1,030 (14.4)	710 (68.9)	274 (26.6)	46 (4.5)
	Somali	803 (11.2)	611 (76.1)	168 (20.9)	24 (3.0)
	Benishangul-Gumuz	576 (8.0)	297 (51.6)	257 (44.6)	22 (3.8)
	SNNP	891 (12.4)	462 (51.9)	391 (43.9)	38 (4.3)
**Variables**	**Categories**	***n*** **(%)**	***n*** **(%)**	***n*** **(%)**	***n*** **(%)**
	Gambela	533 (7.4)	288 (54.0)	200 (37.5)	45 (8.4)
	Harari	410 (5.7)	145 (35.4)	180 (43.9)	85 (20.7)
	Dire Dawa	384 (5.4)	84 (21.9)	201 (52.3)	99 (25.8)
	Addis Ababa	373 (5.2)	22 (5.9)	195 (52.3)	156 (41.8)
Access to media	No	4,636(64.6)	2,966 (64)	1,429 (30.8)	241 (5.2)
	Yes	2,538 (35.4)	771 (30.4)	1,254 (49.4)	513 (20.2)

### Adequacy of prenatal care

**Figure 1:**
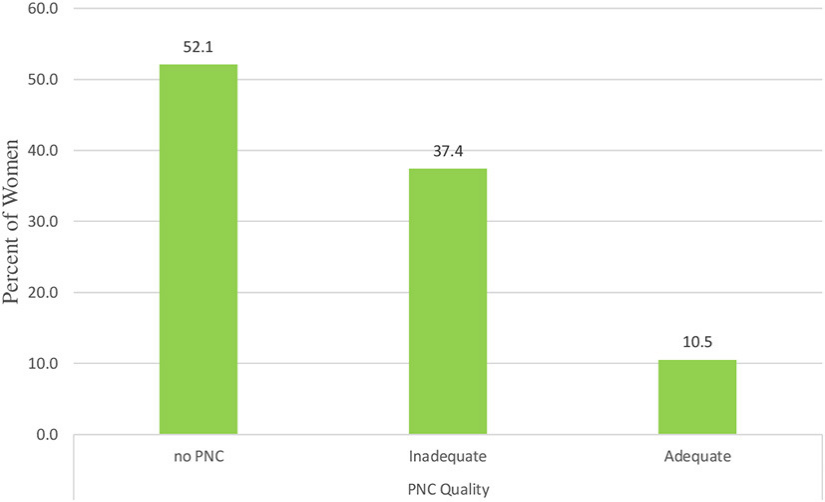
Distribution of the quality of prenatal care services during the most recent pregnancy.

**Figure 2:**
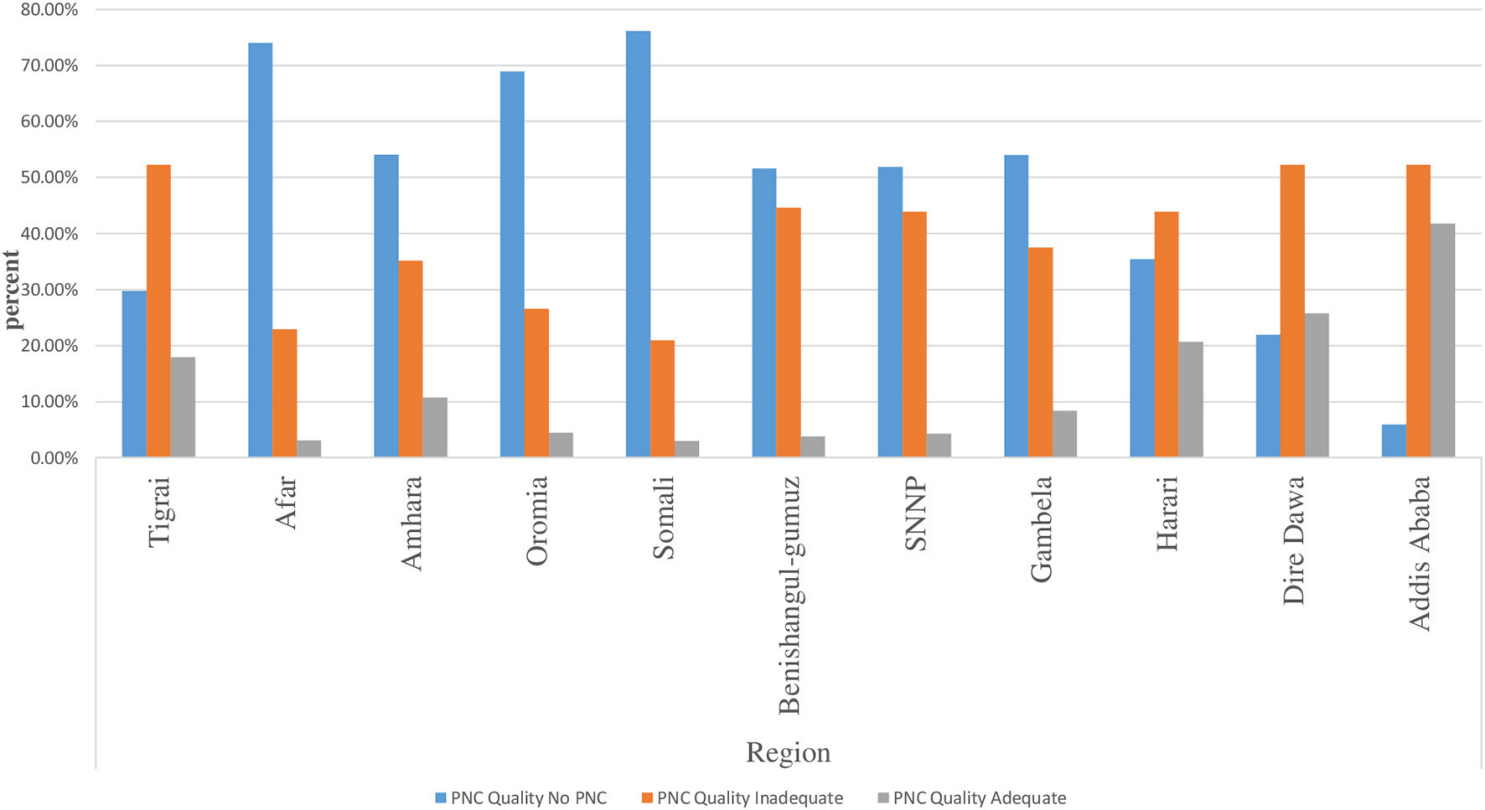
The distribution of prenatal care adequacy by regions.

The distribution of the prevalence of each level of prenatal care adequacy among pregnant women by region is presented in Figure 1. According to the descriptive results, the Afar and Somali regions have the highest prevalence of no PNC, while Addis Ababa has the highest prevalence of adequate PNC. Nearly 52.1% of the participants do not have PNC, while 37.4 and 10.5% of the participants have received inadequate and adequate prenatal care, respectively (Figure 2). In addition to the composite outcome, the study showed that 59.6% received service from a skilled provider, 28% initiated first contact early, 43% had at least four contacts and 25.8% of study participants received at least seven items of recommended components. Only 3.5% of women met the new WHO recommended minimum of eight prenatal contacts.

**Figure 3:**
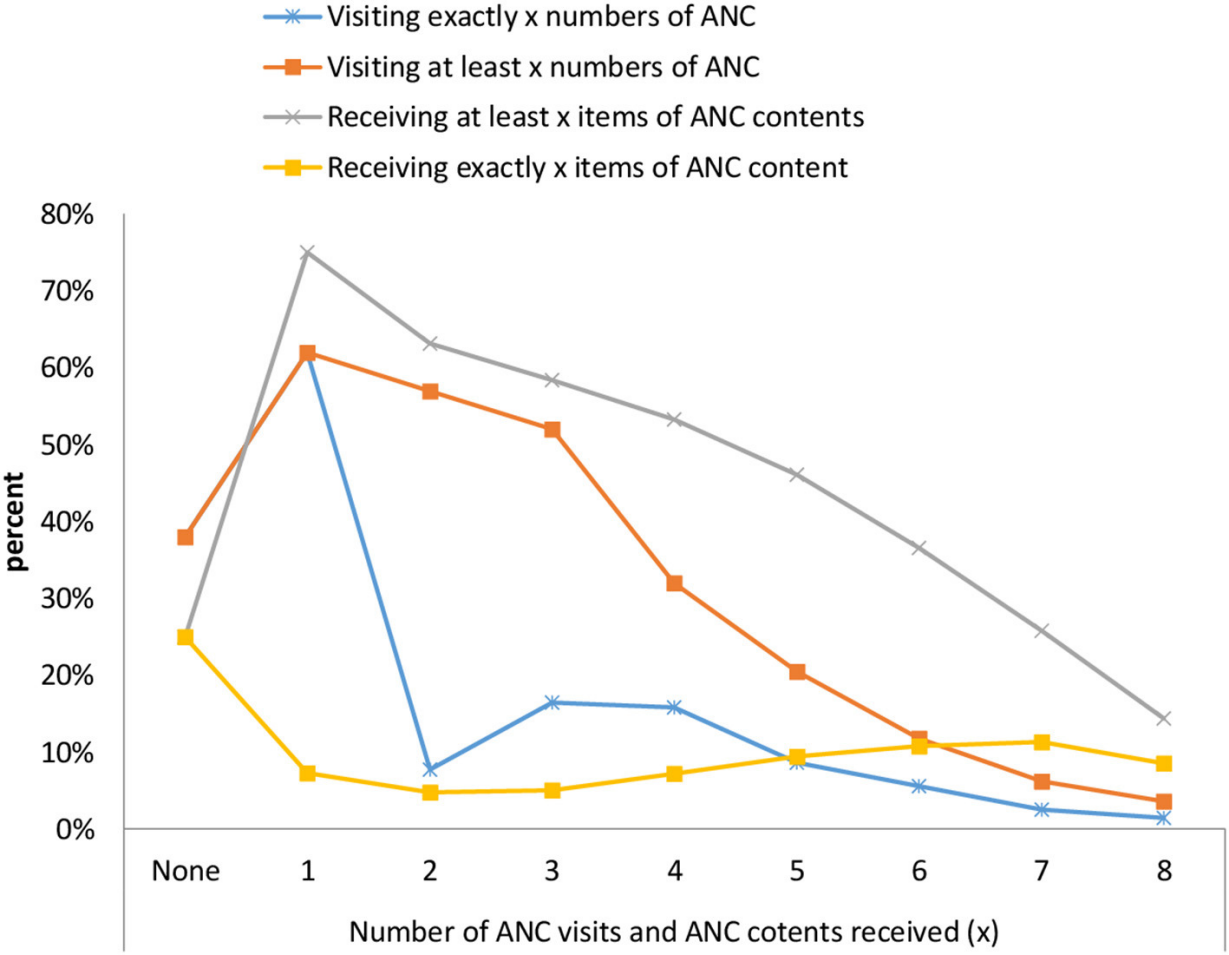
Distribution of the number of ANC contents or items received by the number of prenatal care contacts by women in Ethiopia, interim DHS 2019.

The most common components received by the women during pregnancy were blood pressure measured (88%), followed by blood samples taken (79%), urine samples taken (73%), and nutritional and counseling after testing for HIV/AIDS (70%), while the least common prenatal service item received was treated for intestinal parasites (6%) (Table 2). The study has also outlined that the distribution of prenatal content strictly increases with the number of contacts. About 37% of women who had at least five prenatal contacts during pregnancy had received a minimum of six prenatal contents from skilled providers. Moreover, the mean number of items received was 3.5 ± 2.2 and the mean number of prenatal contacts was 2.63 ± 2.08 per woman (Figure 3).

**Table 2:** The distribution of prenatal care utilization indicators of women, 2019 interim Demographic and Health Survey.

**Dimension**		***n***	**%**
Skilled health care provider	Doctors	311	7.9
	Nurse/midwives	1,834	46.7
	Health officers	197	5
	Health extension workers	550	14
	Traditional birth attendants	8	0.2
	Others Overall skilled provider	28	0.7 59.6
Timing of first ANC contacts	Early (< 3 months)	1,092	28
	4th or 5th month	1,265	32
	6th or 7th month	456	12
	8th + month	8	2
Sufficiency of ANC	No ANC	1,006	26
	1–3 ANC contacts	1,225	31
	At least 4	1,689	43
	At least 8 contacts	314	8
ANC contents	During pregnancy blood pressure measured	3,460	88.1
	During pregnancy urine sample taken	2,902	73.9
	During pregnancy blood sample taken	3,098	78.9
	Counseling about pregnancy complications	1,814	46.2
	Iron supplementations for at least 180 days	1,783	45.4
	Treatment for intestinal parasite during pregnancy	228	5.8
	Counseling after tested for HIV/AIDS during ANC contact	2,714	69.1
	Received health education on birth preparedness	2,109	53.7
	Nutritional counseling	2,788	71
	Received two or more doses of tetanus toxoids vaccine Received at least seven items	1,669	42.5 25.8

### Result of multilevel ordinal logistic regression

#### Model fit statistics

Initially, we started with all the candidate predictors included in the initial model and then applied backward elimination techniques to arrive at the final model. As the Brant test of parallel odds assumption showed that proportional odds appeared to have held constant across all cut-off points of prenatal care adequacy status for the final model (Chi-square = 4.53, *p-*value = 0.77), the proportional odds model was fitted to the data. In the random effects, the results of the null model indicated that there was statistically significant variability in the odds of the adequacy of prenatal care, with a region variance of 0.40. The ICC value was 10.9% (95% CI: 4.89, 22.68%), suggesting that 10.9% of the total variability of prenatal adequacy level was attributed to differences between clusters (regions), while the rest was unexplained. Moreover, the MOR was estimated at 1.83 (95% CI: 1.48, 2.54), revealing that if a woman moved to another community with a higher probability of receiving adequate prenatal care, the median increase in her odds of higher levels of adequate prenatal care would be 1.83, with an ICC of 10.9%. Furthermore, model comparison based on deviance (-2LLR) revealed that model IV was the best-fitted model for the data since it has the lowest deviance value (Table 3). The table shows the estimated variance component among regions, showing regional variability in the prevalence of levels of prenatal care adequacy is 0.40, with a standard error of 0.18. The reported likelihood-ratio test (387.84; *p-*value < 0.0001) shows that there is enough variability between regions to favor a mixed-effects ordered logistic regression over a standard ordered logistic regression. Therefore, using multilevel ordinal logistic regression is appropriate for the analysis.

**Table 3:** Factors associated with adequacy status of prenatal care in Ethiopia, Mini DHS 2019.

**Variables and categories**	**Null model**	**Model II**	**Model III**	**Model IV**
		**AOR (95% CI)**	**AOR (95% CI)**	**AOR (95% CI)**
**Individual-level variables**				
**Wealth quantile**				
Poorest		1		1
Poorer		1.86 (1.56, 2.22)		1.76 (1.48, 2.08)
Middle		2.21 (1.84, 2.67)		2.17 (1.81, 2.59)
Richer		2.50 (2.05, 3.05)		2.52 (2.09, 3.056)
Richest		3.54 (2.79, 4.49)		2.62 (2.04, 3.35)
**Woman education**				
No education		1		1
Primary		1.46 (1.26, 1.69)		1.40 (1.22, 1.61)
Secondary		2.04 (1.51, 2.76)		1.68 (1.32, 2.13)
Higher		2.48 (1.48, 2.17)		1.68 (1.19, 2.36)
**Woman age**				
15–19		1		1
20–24		0.79 (0.59, 1.05)		0.86 (0.65, 1.12)
25–29		0.92 (0.69, 1.22)		1.05 (0.79, 1.39)
30–34		0.89 (0.66, 1.19)		0.99 (0.73, 1.36)
35–39		0.78 (0.58, 1.07)		0.96 (0.69, 1.34)
40–44		0.67 (0.47, 0.95)		0.85 (0.59, 1.25)
45–49		0.72 (0.45, 1.14)		0.95 (0.59, 1.54)
**Access to mass media**				
No access		1		1
at least once a week		1.28 (1.06, 1.56)		1.39 (1.17, 1.64)
**Current pregnancy desired**				
Then		1		1
Later		0.95 (0.79, 1.12)		0.92 (0.79, 1.07)
Not at all		0.68 (0.54, 0.88)		0.69 (0.54, 0.87)
**Spouse education**				
No education		1		1
Primary		1.28 (1.11, 1.47)		1.23 (1.08, 1.39)
Secondary		1.78 (1.42, 2.24)		1.64 (1.35, 1.99)
Higher		1.43 (1.06, 1.92)		1.66 (1.29, 2.11)
**Who decides respondent's healthcare**				
Respondent alone		1		1
Respondent and spouse		1.62 (0.51, 5.19)		1.19 (1.02, 1.86)
Spouse alone		0.83 (0.68, 1.01)		0.84 (0.70, 1.01)
**Perceived Distance to health facility**				
A big problem		1		1
Not a big problem		1.26 (1.08, 1.46)		1.18 (1.03, 1.36)
Health insurance				
No		1		1
Yes		1.45 (1.11, 1.91)		1.69 (1.02, 2.28)
Birth interval				
First Birth		1		1
< 36 months		1.07 (0.93, 1.23)		1.55 (0.86, 2.80)
≥36 months		0.36 (0.22, 0.60)		0.66 (0.33, 0.96)
**Birth order**				
First		1		1
Second		1.06 (0.77, 1.45)		1.04 (0.73, 1.49)
Third		0.41 (0.25, 0.67)		0.21 (0.12, 0.29)
Fourth or higher		0.67 (0.53, 0.89)		0.02 (0.004, 0.03)
**Community level variable**				
**Residence**				
Urban			1	1
Rural			0.76 (0.60, 0.96)	0.69 (0.56, 0.87)
**Community level poverty**				
Low			1	1
High			1.68 (1.23, 2.29)	1.01 (0.92, 1.11)
**Community illiteracy**				
Low			1	1
High			0.73 (0.17, 0.98)	0.32 (0.12, 0.53)
**Community level media use**				
Low			1	1
High			1.21 (1.02, 1.43)	2.13 (1.42, 4.12)
**Community level women autonomy**				
Low			1	1
High			1.23 (1.06, 1.42)	1.89(1.14–2.23)
/cut1	0.28 (0.16, 0.52)	−0.56 (−1.05, −0.09)	−0.79 (−1.27, −0.31)	−0.24 (−0.66, 0.18)
/cut2	0.33 (−0.26, 0.92)	1.18 (0.70, 1.67)	0.92 (0.43, 1.41)	2.54 (2.11, 2.96)
Random effect analysis result				
Community level variance	0.47 (0.20, 1.13)	0.32 (0.13, 0.77)	0.49 (0.21, 1.17)	0.40 (0.17, 0.97)
LR-test	Chi=387.84 (*p*-value ≤ 0.0001)			
ICC	0.109 (0.489, 0.227)			
MOR	1.83 (1.48, 2.54)			
LLR	−6,774.63	−5,862.54	−3,900.31	−3,881.67
Deviance (-2LLR)	13,549.26	11,725.08	7,800.62	7,763.34

LR, Likelihod Ratio; LLR, Log-Likelihood Ratio; ICC, Intra Class Correlation; MOR, Median Odds Ratio; AOR, Adjusted Odds Ratio.

#### Factors associated with adequacy level of prenatal care service utilization

The odds of receiving adequate prenatal care for rural dwellers compared to urban dwellers, an adjusted odds ratio (AOR) = 0.69 with a 95% CI (0.56, 0.87). Regarding education, the odds of receiving adequate prenatal care were estimated to be AOR = 1.40 with 95% CI (1.22, 1.61) for primary, AOR = 1.78 with 95% CI (1.32, 2.19) for secondary and AOR = 1.68 with 95% CI (1.19, 2.36) for a higher level of education. The odds of receiving adequate prenatal care were estimated to be AOR = 1.23 with 95% CI (1.1.08, 1.39) for primary, AOR = 1.6.36 with 95% CI (1.35, 1.99) for secondary, and AOR = 1.66 with 95% CI (1.29, 2.11) for higher education compared to an educated spouse.

For those who wanted no more children, the odds of receiving an adequate level of prenatal care were AOR = 0.69 with 95% CI (0.54, 0.87) compared to those whose pregnancy was wanted. Likewise, for respondents who have access to mass media at least once a week, the odds of receiving adequate prenatal care were estimated to be AOR = 1.39, with a 95% CI (1.12, 1.64). The odds of receiving a high quality of prenatal care instead of a low or inadequate level, for respondents from the middle wealth quantile and the highest wealth quantile compared to the lowest wealth quantile, were estimated to be AOR = 2.17 with 95% CI (1.81, 2.59) and AOR = 2.612 with 95% CI (2.01, 2.35), respectively.

Similarly, the odds of receiving adequate prenatal care were higher [AOR = 1.69, 95% CI (1.02, 2.28)] for women who had covered health insurance compared to those who had no covered health insurance. The odds of receiving adequate prenatal care were estimated to be AOR = 1.18 with 95% CI (1.03, 1.36) for respondents near a health facility compared to those who were reported a long distance from the health center. Further, women in communities with high media use [AOR = 2.13, 95%CI (1.42, 4.12)] and high autonomy [AOR = 1.89, 95%CI (1.14–2.23)] are more likely to receive an adequate level of prenatal care as compared to women in communities with low media use and low autonomy, respectively. Women in communities with high illiteracy [AOR = 0.32, 95%CI (0.12, 0.53)] are less likely to receive an adequate level of prenatal care as compared to women in communities with low illiteracy (Table 3).

## Discussion

In this study, a single composite index of prenatal care adequacy was computed based on skilled health care providers (doctors, nurses, midwives, and health officers), timeliness, the sufficiency of prenatal care and appropriateness of prenatal care contents received recoded into ordinal outcome. A multilevel mixed effects ordinal logistic model (with the logit link function and a robust variance estimator for the standard error) was fitted to the data to identify significant determinants of utilizing an adequate level of prenatal care. Significant factors that are associated with the adequacy of prenatal care services received consist of the place of residence, wealth quantile, woman and spouse's level of education, access to mass media, wanted pregnancy, and distance to the health center.

This study found that place of residence significantly affected the level of adequacy status of prenatal care a pregnant woman had received. This finding is consistent with findings from studies in Ethiopia that found that urban women are more likely to start their first antenatal care early and receive sufficient care (15, 18, 23, 24, 37), Bangladesh (28) and Nigeria (19, 25, 27) which found that the odds of poor quality prenatal care were higher among rural women. This is attributable to poor infrastructure and health facilities with a lack of skilled health care quality in rural areas. People in rural Ethiopia and lower wealth households also face structural problems like the need to travel long distances to care providers and high costs that must be addressed. Furthermore, the majority of caregivers in rural Ethiopia are health extension workers, and there aren't enough professions. The study further indicated that Ethiopian women who had access to mass media (either listening to the radio or watching television at least once a week) were more likely to receive an adequate level of prenatal care from a skilled provider. This result complies with findings from previous studies from Ethiopia, Nigeria, and Bangladesh (25, 27, 28, 37, 38). A possible explanation is that women with access to the media can have more access to information about the health benefits of early starting, prenatal care, and receiving sufficient services from a skilled provider for their child and their health during and after pregnancy (39).

This study identified maternal education as a potential factor associated with prenatal care adequacy. Compared with a woman with no education, a woman with at least primary maternal education has received a more adequate level of prenatal care. Several previous studies from Ethiopia (15, 18, 23, 24, 37, 38), Brazil (40), Nigeria (19, 25, 27), Kenya (41), Ghana (26) and Nepal (42) supported this finding, indicating that women with higher education were more likely to receive an adequate level of prenatal care as compared with women who had lower or no education, indicating that women's education is an essential factor affecting the quality of prenatal care services received. As educated women have a better knowledge of health and nutrition, they are more conscious of their health and children's health (43). Not only the woman's education but also her spouse's education was significantly associated with receiving adequate prenatal care from a skilled provider, and women whose spouse had at least primary education had a higher chance of receiving adequate prenatal care. Other studies reported similar findings (27, 37, 42). Spouses with a higher level of education know better about the benefits of influence on joint decision-making among couples concerning health-seeking at the time of pregnancy (44).

This study found a significant association between household wealth quintile and prenatal care adequacy level status. Compared to a woman from a poor wealth quintile, a woman from a middle or higher wealth quintile is more likely to have a higher adequacy level and worse undernutrition status. Prior studies support the results of this study (15, 19, 23, 27, 28, 37, 38, 41, 42). This could be explained by the fact that increased income improves health care needs, which in turn improves the adequacy of health care intake. Because the household wealth quintile is an important factor in poor prenatal care status, free services or financial support are essential for poor women during the prenatal period to improve the quality of prenatal care received from a skilled provider. In this study, covered health insurance was found to be positively correlated with the adequacy status of prenatal care. Findings from similar studies in Gabon revealed that, compared to women who had no health insurance, women with covered health insurance had significantly higher odds of receiving adequate prenatal care (45).

Similar to findings from previous studies (28, 42), this study also identified current pregnancy desire as a determinant factor of prenatal care adequacy status. If a woman's current pregnancy is not wanted at all, she has a lower chance of receiving adequate prenatal care than if it is wanted. The study further indicated that the decision power of respondent' health care is significantly associated with prenatal care adequacy status. Accordingly, a woman who jointly decides on respondent health care has a higher odds of receiving adequate prenatal care, which complies with findings from previous studies in Ethiopia (24, 44), and Pakistan (45). This could be explained by the fact that joint discussions might help both women and their spouses arrive at a consensus decision on when, where, and how to cover the cost of health care services.

Findings from this study showed that the longer the distance to health facilities, the lower the odds of receiving adequate prenatal care. Findings from prior studies in Ethiopia and elsewhere (16, 25, 27) identified distance to a health facility as a significant determinant of prenatal care utilization. This study found that compared to the first birth, the odds of receiving adequate prenatal care were lower among women who had at least 36 months of the previous birth interval, a conclusion that complies with findings from previous studies (27).

## Conclusion

This study aimed to identify the associated factors of prenatal care service utilization in Ethiopia using a single composite index as a response variable computed from the skilled health care providers (doctors, nurses, midwives, and health officers), timeliness, and sufficiency of prenatal care and appropriateness of prenatal care contents received. Assessing compliance with the WHO recommended standard guidelines, about 43% met the old minimum of four prenatal contacts, while only 3.5% met the new WHO recommended minimum of eight prenatal contacts. The prevalence of adequate prenatal care was lower (10.5%). The multilevel mixed effects model revealed that place of residence, exposure to mass media, education level of woman and spouse, wealth quintile, current pregnancy desired, who decides the respondent's health care, distance to the health facility, health insurance coverage, and birth interval were significantly related to prenatal care adequacy status.

A large majority of pregnant women received inadequate prenatal care and the need for proven health care interventions such as the expansion of health facilities and health education in all policies aiming to reduce maternal and neonatal deaths. Traveling long distances to care providers has high costs that must be addressed, particularly in rural areas and among lower-income households. Further financing of maternal care through covered health insurance and free maternal care for all women might be encouraged through interventions.

## Limitations of the study

Recall bias might affect the data since data was mothers' recall of past events, since the recall period is long (five years preceding the survey). The other limitation of the study was that some important possible factors that could affect adequacy of prenatal care are missed due to incompleteness of information. Further, it is difficult to determine causation from cross-sectional studies. Moreover, Joint decision making by both partners does not necessarily mean that the woman was given equal decision-making.

## Data availability statement

The original contributions presented in the study are included in the article/supplementary materials, further inquiries can be directed to the corresponding author/s.

## Ethics statement

This study used publicly available Ethiopian Mini Demographic and Health Survey (EMDHS) data. Permission was obtained from the Measure DHS program to access and analyze the data. During EMDHS data collection, Informed consent was taken from each participant, and all identifiers were removed and the confidentiality of the information was maintained. Written informed consent for participation was not required for this study in accordance with the national legislation and the institutional requirements.

## Author contributions

The author has participated in conception and design, or analysis and interpretation of the data, drafting the article or revising it critically for important intellectual content, and approval of the final version.

## Conflict of interest

The author declares that the research was conducted in the absence of any commercial or financial relationships that could be construed as a potential conflict of interest.

## Publisher's note

All claims expressed in this article are solely those of the authors and do not necessarily represent those of their affiliated organizations, or those of the publisher, the editors and the reviewers. Any product that may be evaluated in this article, or claim that may be made by its manufacturer, is not guaranteed or endorsed by the publisher.
